# Syllabus of the Obstetrical Lectures in the Medical Department of the University of Pennsylvania

**Published:** 1890-05

**Authors:** 


**Syllabus of the Obstetrical Lectures in the Medical Department of the University of Pennsylvania.** By RICHARD C. NORRIS, A. M., M.D., Demonstrator of Obstetrics. Philadelphia: W. B. Saunders. 913 Walnut street.

This syllabus is designed to overcome the difficulty of accurate note-taking, being especially adapted to the course of obstetrics, given by Professor Hirst, at the University of Pennsylvania. Like all works of this character, it is of use only to students who are cramming for examinations, and is of little value to the profession. This outline of lectures given by Prof. Hirst shows the wide range of subjects comprehended in the course of obstetrics given at that excellent institution.

We notice much that would not be adapted to other schools, however valuable they may be for the course to which they were given. This criticism would be applicable to the efforts of every lecturer. Circumstances, locality, etc., determine the extent and character of the lecture courses everywhere. On the whole, the work will fill its purpose.

